# Identifying Hypoxia in a Newborn Piglet Model Using Urinary NMR Metabolomic Profiling

**DOI:** 10.1371/journal.pone.0065035

**Published:** 2013-05-31

**Authors:** Christopher Skappak, Shana Regush, Po-Yin Cheung, Darryl J. Adamko

**Affiliations:** 1 Department of Pediatrics, Faculty of Medicine and Dentistry, University of Alberta, Edmonton, Alberta, Canada; 2 Department of Pharmacology, Faculty of Medicine and Dentistry, University of Alberta, Edmonton, Alberta, Canada; 3 Department of Medicine, Faculty of Medicine and Dentistry, University of Alberta, Edmonton, Alberta, Canada; 4 Department of Pediatrics, Faculty of Medicine, University of Saskatchewan, Saskatoon, Saskatchewan, Canada; Instituto de Investigación Sanitaria INCLIVA, Spain

## Abstract

Establishing the severity of hypoxic insult during the delivery of a neonate is key step in the determining the type of therapy administered. While successful therapy is present, current methods for assessing hypoxic injuries in the neonate are limited. Urine Nuclear Magnetic Resonance (NMR) metabolomics allows for the rapid non-invasive assessment of a multitude breakdown products of physiological processes. In a newborn piglet model of hypoxia, we used NMR spectroscopy to determine the levels of metabolites in urine samples, which were correlated with physiological measurements. Using PLS-DA analysis, we identified 13 urinary metabolites that differentiated hypoxic versus nonhypoxic animals (1-methylnicotinamide, 2-oxoglutarate, alanine, asparagine, betaine, citrate, creatine, fumarate, hippurate, lactate, N-acetylglycine, N-carbamoyl-β-alanine, and valine). Using this metabolomic profile, we then were able to blindly identify hypoxic animals correctly 84% of the time compared to nonhypoxic controls. This was better than using physiologic measures alone. Metabolomic profiling of urine has potential for identifying neonates that have undergone episodes of hypoxia.

## Introduction

Every delivery of an infant is a physiologically stressful event and hypoxemic episodes occur. Current methods for evaluating the level of damage from a hypoxic episode are limited, and there is a clinical need for a precise assessment of a neonate’s hypoxic state to improve patient care [1]. Neuroprotective therapies used to treat hypoxic episodes are successful but must be applied within a relatively short time period [2]. The clinical methods used to assess neonatal hypoxic episodes include Apgar scores, serum lactate levels, serum acid-base deficits, electroencephalography (EEG) recordings, and magnetic resonance imaging (MRI). While each of these tests offer valuable information that can be clinically relevant in predicting some outcome of neonatal hypoxia, none of them have been proven to have the sensitivity needed to affect treatment. The lack of ability to identify acute hypoxic changes in the neonate leaves neonatologists without direction when it comes to implementing early therapies such as hypothermia treatment [3]. Development of a diagnostic tool able to rapidly assess the level of hypoxic damage experienced by a neonate would allow therapies to be initiated sooner, theoretically preventing long-term neurological deficits.

Nuclear magnetic resonance (NMR) based metabolomics is a powerful tool that offers the opportunity to investigate biochemical changes in response to a disease state and/or injury. The utility of this diagnostic technique is now being explored in the field of neonatal medicine [4]. One critical area being investigated is the potential ability for metabolomics to identify and quantitate damage and the extent of recovery from periods of neonatal asphyxia [5]–[7]. Currently animal models of neonatal hypoxia offer precise physiological measurements that can be directly compared to acute changes in the urine metabolome in order to establish a model of neonatal hypoxic injury [8], [9]. In this study we used NMR metabolomics in conjunction with physiological measurements to establish a metabolomic profile of neonatal hypoxia-reoxygenation (H-R).

## Methods

### Ethics Statement

All experiments were conducted in accordance with the guidelines of Canadian Council of Animal Care (2001) and approved by the Animal Care and Use Committee: Health Sciences, University of Alberta (ACUC: HS Protocol **#**183/10/10B).

### Surgical Preparation for All Animals

Male newborn Yorkshire-Landrace piglets 1–3 day of age weighing 1.6 to 2.5 kg were used (n = 32). The animal preparation was similar to that described previously [9]. Briefly, anesthesia was initially maintained with inhaled isoflurane (2–3%), which was then switched with fentanyl (0.005–0.05 mg/kg/h), midazolam (0.1–0.2 mg/kg/h) and pancuronium (0.05–0.1 mg/kg/h) once mechanical ventilation was commenced. Oxygen saturation was continuously monitored with a pulse oximeter (Nellcor, Hayward, CA), and heart rate and blood pressure were measured with a 78833B monitor (Hewlett Packard Co., Palo Alto, CA). Fractional inspired oxygen concentration (FiO_2_) was measured by an oxygen monitor (Ohmeda Medical, Laurel, MD) and maintained at 0.21–0.24 for oxygen saturation between 90 and 97%. Argyle catheters (5F; Sherwood Medical Co., St. Louis, MO) were inserted via the right femoral artery and vein for continuous measurement of mean arterial pressure (MAP) and central venous pressure, respectively. All medications and fluids were administered via the femoral venous catheter. Via a tracheotomy, pressure-controlled assisted ventilation was commenced (Model IV-100, Sechrist Industries Inc., Anaheim, CA) with pressure of 20/4 cm H_2_O at a rate of 18–20 breaths/min. A left anterior thoracotomy was performed to expose the main pulmonary artery. A 6-mm transit time ultrasound flow probe (6SB, Transonic Systems Inc., Ithaca, NY) was placed around the main pulmonary artery to measure the blood flow as a surrogate of cardiac output (CO). The ductus arteriosus was ligated. A 20G Insyte-W angiocatheter was inserted into bladder transcutaneously to drain the urine.

Maintenance fluids during experimentation consisted of 5% dextrose at 10 ml/kg/h and 0.9% normal saline solution at 2 ml/kg/h. The dosages of fentanyl, midazolam and pancuronium were adjusted to maintain minimum body movements throughout the experimental period. Propofol (0.1–0.2 mg/kg/h) was given as needed to maintain anesthesia. The body temperature was maintained at 38.5–39.5**°**C using an overhead warmer and a heating pad.

### Experimental Protocol

After surgery, animals were stabilized for at least 60 min. Piglets were block-randomized into a sham-operated group (ventilation with FiO_2_ of 0.21 without hypoxia for 6 h, n = 15) or a hypoxia-reoxygenation (H-R) group (ventilation for 2 h with an FiO_2_ of 0.10–0.13 using nitrogen and oxygen gas mixture achieving a partial pressure of oxygen [PaO_2_] 20–40 mmHg, n = 17). After hypoxia, the HR piglets were resuscitated with a FiO_2_ of 1.0 for 0.5 h, followed by 0.21 for the last 3.5 h of the experimental period. Blood gases were studied every 30–60 min throughout the experiment. Peak inspiratory pressure (18–25 cm H_2_O) and respiratory rate (12–20 breaths/min) were adjusted in all animals in an attempt maintain normocapnia during experimentation. At the end of the experiment, the piglet was euthanized with an overdose of pentobarbital (100 mg/kg, i.v.).

### Physiological Recordings and Calculations

Hemodynamic parameters (heart rate, MAP and pulmonary artery flow) were recorded at specific predetermined time points at baseline and throughout hypoxia and reoxygenation.

### Statistical Analysis of Physiological Data

All results were expressed as median and inter-quartile range. One- way analysis of variance (ANOVA) tests with Bonferroni correction was used to study the differences among groups. Statistical analyses were performed using Prism 6 (Graphpad software, SanDiego, CA) Significance was set at p<0.05.

### Urine Sample Collection and Preparation

A single urine sample was collected from each piglet at 3 different time points and promptly placed in a freezer (–20°C). Within 1 hour of collection, the urine samples were stored in a –80°C freezer. Samples were analyzed within 6 months of collection we have previously reported that such samples can be stable in the freezer for up to a year. [10].

Urine samples were thawed only once in a biosafety fume hood, and a 630-µL aliquot was removed and placed in a 1.5-mL Eppendorf tube followed by the addition of 70 µL of a reference buffer solution (4.9 mmol/L disodium-2, 2-dimethyl 2-silapentane-5-sulfonate and 100 mmol/L imidazole in deuterium oxide; Chenomx Inc., Edmonton, Alberta, Canada). The sample was vortexed and the pH of each sample was adjusted to 6.7±0.1 by using HCl and NaOH before transferring an aliquot of 600 µL to a standard 5-mm glass NMR tube (Wilmad LabGlass, Wilmad, NJ).

### NMR Spectral Acquisition

The following details describe the type of NMR spectrometer and the precise magnetic pulse sequences used to generate the spectra. All ^1^H-NMR spectra were acquired on a 600-MHz VNMRS spectrometer (Varian Inc., Palo Alto, Calf) equipped with a 5-mm inverse-proton (HX) probe with z-axis gradient coil. One-dimensional ^1^H-NMR spectra were collected at 258C by using the first increment of a 2-dimensional-^1^H,^1^H-NOESY (1-dimensional, 3-pulseNOESY,with a transmitter presaturation delay of 900 ms for water suppression during the preacquisition delay and 100 ms mixing time), and a spectral width of 7200 Hz (phase cycle available on request). The time-domain data points were 64 kilo complex points, acquisition time was 4 seconds, the 908 pulse was 6.8 microseconds, repetition time was 5 seconds, there were 4 steady-state scans, and the number of acquired scans was 32 per free induction decay. The data were apodized with an exponential window function corresponding to a line broadening of 0.5 Hz, 0-filled to 128 k complex points, and Fourier transformed.

### Metabolomic Data Analysis

Spectral identification and quantification of 50 identifiable metabolites was performed by using the Chenomx NMR Suite Professional software package Version 7.1 (Chenomx Inc.). The software contains a database of known metabolites with their referenced ^1^H-NMR spectral resonant frequencies or signatures. These known resonant frequencies were matched to the observed resonant frequencies of the collected spectra, enabling the qualitative and quantitative analysis of metabolites in urine. To account for potential differences in hydration, each metabolite value was standardized to the animal’s measurement of urine creatinine. Partial least squares discriminant analysis (PLS-DA) was performed (SIMCA-P 11, Umetrics, USA), which determines the relationship between the response vector Y (i.e. sham control group versus hypoxemia) and the matrix X (concentration of each metabolite) by simultaneous projections of both Y and X spaces to a plane. Seven-fold internal cross validation was performed. PLS-DA generates a prediction score (0–1) for each animal based on the value of the metabolites (i.e., scores <0.5 would be predicted to be sham control animals versus hypoxic animal >0.5). This process identifies the metabolites whose concentrations differed significantly between groups of animals. As might be expected, most metabolites measured do not differ greatly between groups. In contrast, the greater the consistent difference in metabolite concentration between groups, the more important a metabolite becomes. Metabolites of low significance are detrimental to model accuracy and should be removed. We removed all but 13 metabolites using a blinded test set (see Results below).

## Results

### Physiologic Data Alone cannot not Accurately Predict Hypoxemia

**Figure 1:**
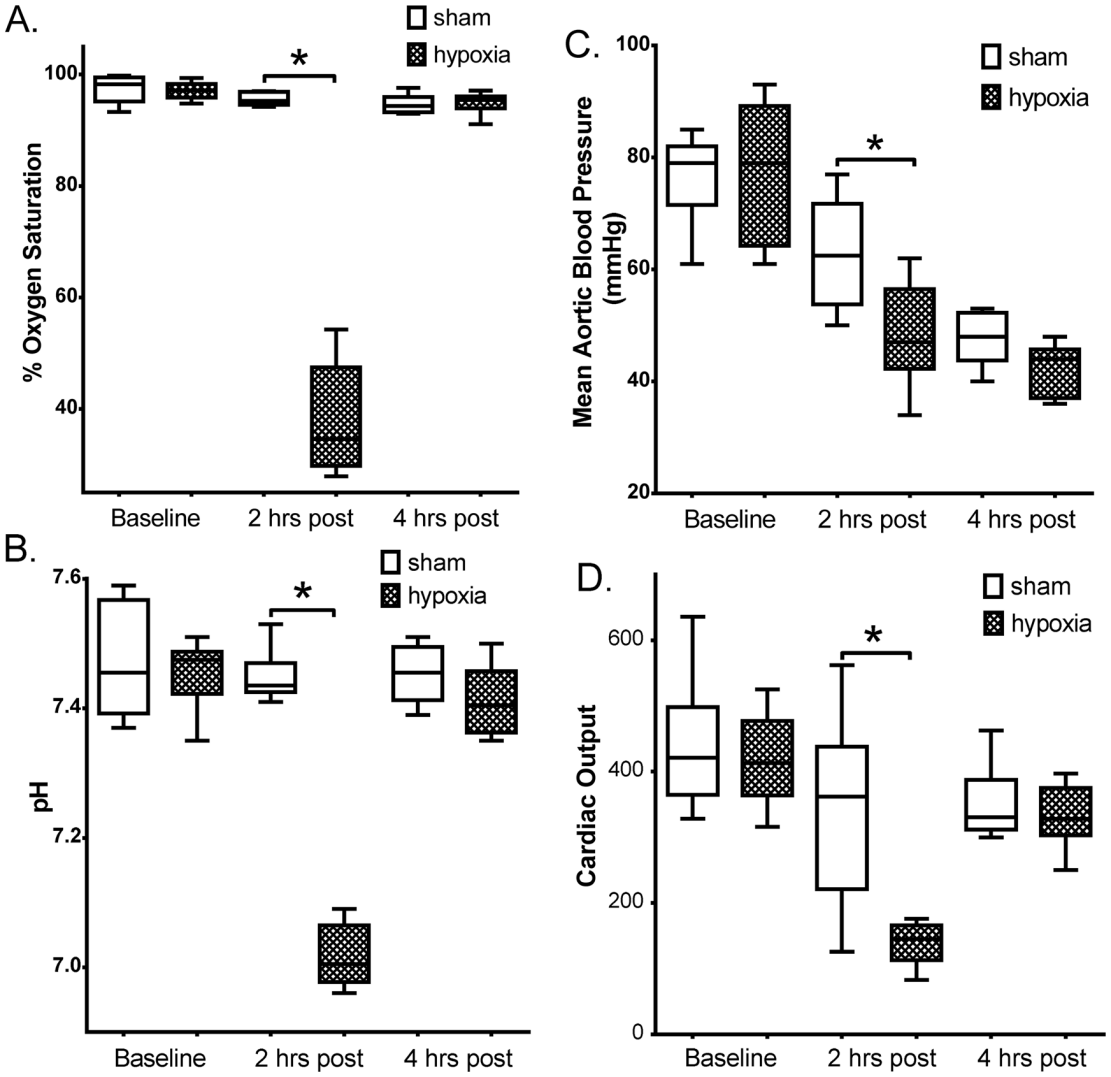
Physiological effects of hypoxia in newborn piglets. Temporal changes in (A) oxygen saturation (% O_2_), (B) blood pH, (C) mean arterial pressure (mmHg), and (D) cardiac output between hypoxia (n = 7) and sham (n = 6) treated animals. *P<0.05 Sham vs. Hypoxia for corresponding time point.

Both groups of animals were subjected to the same invasive surgical preparation in order to acquire the required physiological measurements. As a result, all animals showed some physiologic stress and a decline in all parameters measured by the end of experiment (Figure 1, arterial oxygen saturation, pH, MAP, and CO). There were no statistically significant differences in these measurements between groups at baseline post-surgical set-up. As might be expected, after 2 h of hypoxia, the H-R animals had statistically lower values for all physiologic parameters measured. In contrast, after 4 hours of recovery at the 6 h period, the physiologic measurements in H-R animals returned to values similar to that of the non-hypoxia control animals.

### Despite Normal Physiology, the Urine NMR Profile of Hypoxic Challenged Animals Differs Compared to Non-hypoxic Sham Controls

**Figure 2:**
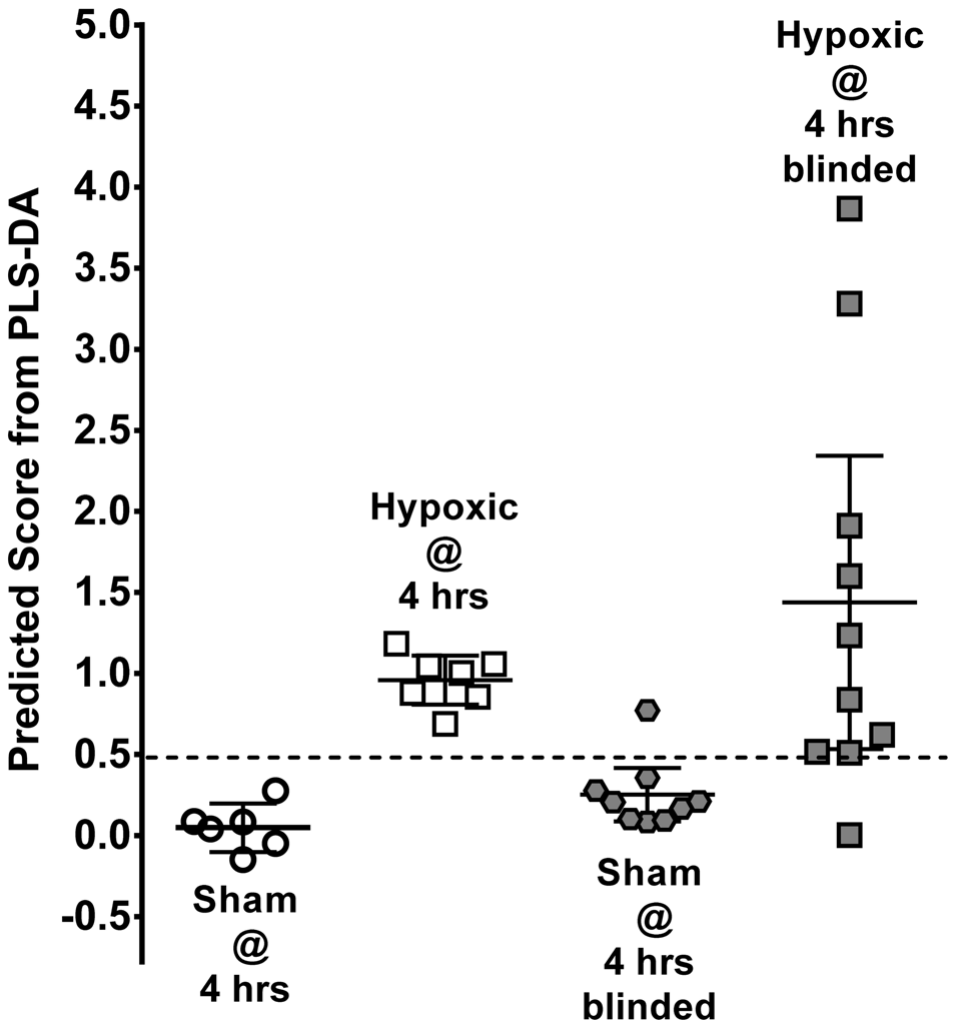
Differentiating hypoxic animals vs. sham treated animals. The PLS-DA algorithm separates groups of data based on a score of 0–1; in this case a value closer to zero indicates no hypoxemia (sham, n = 6) and above 0.5 indicates hypoxemia (n = 7). Illustrated are the PLS-DA prediction scores for each animal including blinded test groups. Error bars represent medians and interquartile ranges.

**Figure 3:**
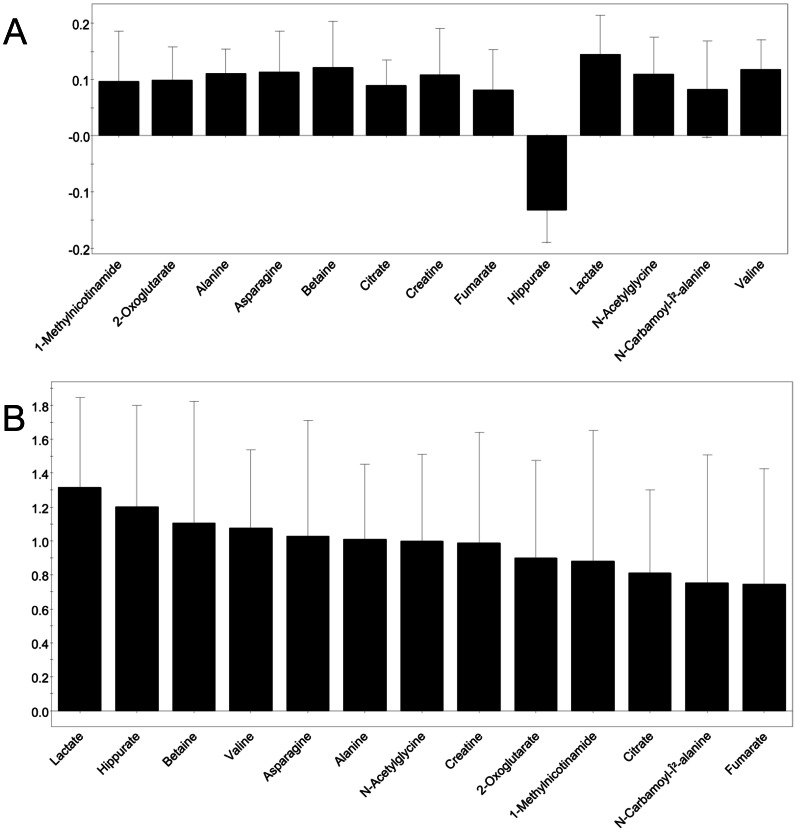
The metabolomic model of hypoxic vs. sham treated animals. PLS-DA analysis of urine from hypoxic versus sham treated animals was based on differences in metabolites between groups shown as the Coefficient of Variation (CoV) plot (A). The importance of each metabolite within the model is shown as the Variability of Importance (VIP) plot (B).

While urine samples were collected from both groups at baseline, 2, and 6 hours, the 6-hour time-point was chosen to create the diagnostic metabolomic model. We thought this time period would best replicate the clinical scenario of an infant post-hypoxic insult. The concentrations of 50 metabolites were measured in the urine samples (Chenomx, Edmonton, AB) and standardized to their respective creatinine level. Based on these values for each group of animals, PLS-DA created a model of separation between hypoxemic (n = 7) and sham treated animals (n = 6). As would be expected, many of the metabolites excreted in the urine did not differ greatly between groups, and leaving metabolites of low importance rendered the metabolite model less accurate, including the possibility of false positive results. To remove these metabolites and improve accuracy, we used a test set of urine samples from sham treated animals not part of the model (n = 9). We removed as many metabolites as possible while still maintaining the best possible correct classification score of blinded sham treated animals (PLS-DA prediction score <0.5; 8 of 9 animals correct; figure 2). The final model for PLS-DA separation of non-hypoxic animals from hypoxic animals at 6-hours consisted of 13 metabolites. The model used one component giving an R2 = 0.911 and Q2 = 0.892. The differences in concentration of these metabolites between groups are shown as the Coefficient of Variation Plot (Figure 3A), and the ranking of metabolite importance for separation is shown as the Variability of Importance Plot (Figure 3B). The final metabolites chosen and their average concentrations for each animal group are shown in Table 1. To validate the proposed model as a diagnostic tool for hypoxic insult, we entered the concentrations of metabolites from HR animals not originally part of the modeling exercise (n = 10). The PLS-DA model correctly diagnosed the blinded hypoxic samples with 90% accuracy (9/10 samples).

**Table 1:** Average concentrations and inter-quartile ranges of urinary metabolites in hypoxia and sham treated newborn piglets used to generate the metabolomic model (in mmol of metabolite/mmol creatinine).

Metabolite	Sham (IQ range)n = 6	Hypoxia (IQ range)n = 7
Alanine	0.211 (0.137)	0.639 (0.364)
Asparagine	0.054 (0.030)	0.038 (0.007)
Betaine	0.116 (0.093)	0.705 (0.607)
Citrate	0.428 (0.239)	0.887 (1.412)
Creatine	0.030 (0.022)	0.166 (0.207)
Fumarate	0.062 (0.044)	0.127 (0.134)
Hippurate	1.375 (0.577)	0.685 (0.364)
Lactate	0.457 (0.359)	7.946 (5.630)
1-Methylnicotinamide	0.103 (0.030)	0.175 (0.178)
N-Acetylglycine	0.070 (0.081)	0.180 (0.157)
N-Carbamoyl-β-alanine	0.347 (0.282)	0.615 (0.489)
2-Oxoglutarate	0.265 (0.212)	0.465 (0.421)
Valine	0.007 (0.005)	0.038 (0.014)

IQ range = Q3-Q1.

## Discussion

Identifying neonatal hypoxemia and determining if and when to initiate potential neuroprotective therapy remains a challenging issue based on current diagnostic techniques. Apgar scores are affected by a poor method of assessing neonatal asphyxia [11]. This is due to a number of variables such as maternal sedation and analgesia, and underlying cardiovascular or neurological conditions in the neonate [12]. Measuring serum lactate levels and base deficits do show some positive predictability in identifying neonates that have experienced severe hypoxia, but unfortunately are not reliable when assessing mild to moderate hypoxia or in conditions like septicemia [13], [1], [14]. Predicting the level of developmental delay is based around neurological examinations and the use of EEG recordings [15], [16]. A recent examination of the use of EEG in staging neonatal hypoxic episodes and predicting severe outcomes found that EEG performed within 6 hours of a hypoxic birth did not predict long-term outcomes involving disability and death [17]. The use of MRI to evaluate hypoxic brain injury in neonates has also been established, however MRI requires an anesthesia for the infant and is not able to identify and evaluate early hypoxic changes [18], [19]. The limitations of the current diagnostic methods leave a large gap in the ability of physicians to accurately diagnose and assess the degree of hypoxia experienced by an infant. An ideal diagnostic to mitigate this deficiency would be one that is non-invasive, available at the bedside in the NICU, and would provide an accurate assessment of the patient’s condition.

NMR metabolomic analysis of urine from hypoxemic neonates offers a non-invasive solution. It can be used to rapidly identify and quantify the compounds that could provide the basis of a bedside urinary diagnostic test. Further, in contrast, to measuring just one variable like lactate, NMR metabolomics studies a large number of variables covering multiple metabolic pathways. Our report identified a combination of 13 metabolites that could be used to identify a hypoxemic episode in the animal model. Similar to other studies using NMR and mass spectrometry analysis of urine in fetal piglet models of hypoxia, we confirmed the presence of 6 compounds including, alanine, citrate, creatine, fumarate, lactate, succinate, and valine [5], [6]. In addition, we found the following compounds were uniquely identified in our NMR analysis: 1-methylnicotinamide, 2-oxoglutarate, asparagine, betaine, hippurate, N-acetylglycine, and N-carbamoyl-β-alanine.

Many of the metabolites identified were directly related to cellular energy levels and metabolism. Not surprisingly, lactate, which was a key metabolite, is a product of anaerobic respiration and is known as an early marker of neonatal hypoxia in humans [20]. Citrate, an important intermediate in energy metabolism particularly in the citric acid cycle, was increased. This is also reported in the CSF of hypoxic fetal sheep [21]. Mitochondria appear to have up to a 40% decrease in the ability to use citrate and malate under hypoxic conditions [22]. Creatine and one of its products phosphocreatine are also important components of energy metabolism in muscle. Rises in creatine in the absence of phosphocreatine may be indicative of an impaired metabolism due to an energy-depleted system [23]. Creatine has also been found to have a neuro-protectant role as an anti-oxidant in hypoxic chick spinal cord neuron cultures [24]. Fumarate and 2-oxogluterate are components of the citric acid cycle important for energy metabolism [25]. N-acetylglycine is an N-acytylated derivative of glycine that is normally broken down by an ATP dependent pathway; high levels of this molecule may indicate an energy-deprived state [26]. Together these metabolites reflect the major impact mild to moderate hypoxia can have on aerobic respiration and cellular metabolism.

The remaining metabolites identified were likely the result of hypoxia’s effects on inflammation and dysregulation of amino acids. Many of these metabolites relate to the body’s ability to self-limit inflammation and cellular dysfunction that accompany ischemic injury. Alanine levels are known to increase in the cerebral spinal fluid (CSF) of fetal sheep during a hypoxic episode, and they remain high for 2 hours following the episode before gradually decreasing [21]. The initial increase then gradual decrease may be due to the consumption of alanine to make the excitatory amino acid aspartate, which can also have damaging effects on the brain [27]. Betaine, a trimethylglycine is known to act as a protective osmolyte and act as a methyl donor [28]. Betaine and methyl donor insufficiency has been associated with metabolic disorders and impaired fetal development in humans [28]. High levels of betaine are reported to decrease the inflammatory response of adipocytes stressed under hypoxic conditions [29]. Hippurate an excretion product of odd-chain fatty acid breakdown was found to decrease in hypoxia [25]. Hippurate was also shown to be decreased in the urine of human stroke patients with-in 72 hours of an ischemic event, this is believed to be related to folic acid deficiency and hyperhomocysteinemia [30], [31]. N-carbomyl-β-alanine has been found to play a role in the protection of the liver against hypoxic injury in a rat model via aiding in ion homeostasis [32]. 1-Methylnicotinamide (1MNT), a metabolite of nicotinamide was found to have anti-inflammatory properties [33]. High dose administration of 1MNT has been shown to inhibit a pro-inflammatory enzyme (matrix metalloproteinase 9) in the brains of neonatal rats during the acute phase of a hypoxic episode. High levels of this metabolite may be an endogenous anti-inflammatory agent of the brain [34]. Valine, an amino acid, was found to be increased in the urine of our hypoxic animals, this corresponds with previous studies that demonstrated increased release of valine from dog brain tissue in-vivo over a 30 minute episode of hypoxia [35].

It is important to note that no single metabolite could diagnose all animals correctly. For example while critical, lactate was also elevated in some non-hypoxic animals. Sham animals did undergo a similar invasive surgical protocol but without hypoxemia. For an animal to be diagnosed as hypoxic, it required a combination of metabolites. At the 6-hour period, the hypoxic animals had time to recover, and both groups had similar levels of oxygen saturation, pH, MAP and CI. This might mimic the real world experience of a birth. We suggest that to have accuracy at diagnosis a combination of variables will be needed. Metabolomics provides this option. Ideally in the future this profile will be confirmed in human neonates, and a non-NMR based point of care diagnostic could be developed from this urinary metabolomic profile. The noninvasive nature of urine makes this test applicable to non-tertiary care settings where deliveries most often occur. A first urine sample may become very insightful for obstetricians and family doctors performing deliveries. Such metabolomic-based data might warrant the care of a neonatologist.
